# Psychological capital and psychological well-being among caregivers of individuals with alcohol use disorders: a mixed-methods public health study incorporating ayurvedic perspectives

**DOI:** 10.3389/fmed.2026.1836599

**Published:** 2026-08-06

**Authors:** Prerana Jain, Reshma N. S., Sushma Naranappa Salethoor, Shyamasundaran Kulangara, Radhika Kolavarambath, Vasudha Kadikadka Gangadhara

**Affiliations:** 1 Department of Psychiatry, Kasturba Medical College Mangalore, Manipal Academy of Higher Education, Manipal, India; 2 Department of Shalakya Tantra, Amrita School of Ayurveda, Amrita Centre for Advanced Research in Ayurveda, Amrita Vishwa Vidyapeetham, Kollam, India; 3 Department of Samhita, Sanskrit and Siddhanta, Amrita School of Ayurveda, Amrita Centre for Advanced Research in Ayurveda, Amrita Vishwa Vidyapeetham, Kollam, India

**Keywords:** alcohol dependence syndrome, caregivers, Paricaraka/Upasthata, primary health care, psychological capital, psychological well-being

## Abstract

In India’s mental health service delivery, caregivers’ needs are often overlooked despite the substantial burden and stress they experience, particularly when caring for individuals with alcohol use disorders (AUD), which in turn affects patient recovery. Ayurveda conceptualizes treatment as resting on four sub-pillars, one of which is the caregiver, and emphasizes specific caregiver qualities, called *“Parichara”* qualities, essential for favorable treatment outcomes. These include *Buddhiman, Daksha, Anurakta*, and *Shuchi*. These Ayurvedic concepts align with psychological capital (PsyCap), and this psychological capital plays a key role in enhancing the psychological well-being of caregivers. The study aimed to examine predictors of psychological well-being (PWB) among caregivers and to explore the psychosocial factors influencing their wellbeing. A concurrent convergent mixed-methods design was employed utilizing the Compound PsyCap Scale–Revised and Ryff’s PWB Scale among 125 caregivers recruited from two tertiary care hospitals. Socio-demographic variables predicted 37%, and PsyCap predicted an inverse relationship with the PWB. Additionally, a semi-structured interview was conducted with 10 participants, highlight themes related to associated challenges and stressors, as well as psychological factors that aid in facing these challenges. The study findings will equip healthcare professionals with the knowledge needed to enhance caregiver resilience, thereby improving treatment adherence and overall community mental health services for both individuals with AUD and their caregivers.

## 1 Introduction

Alcohol use disorder (AUD) is a chronic medical condition with an impaired ability to regulate alcohol consumption, despite the presence of negative social, occupational, or health-related consequences (1). Alcohol consumption significantly and directly challenges the United Nations’ 2030 Agenda, impacting Sustainable Development Goals (SDGs), specifically SDG 3, health and wellbeing (2). In the context of treating individuals with AUD, caregivers play a crucial role in the recovery process. Literature demonstrates that caregivers of individuals with AUD frequently experience greater levels of objective and subjective burden, encompassing disruptions in daily routines, considerable physical and financial strain, social isolation, and psychological distress (3–5). In India, there has been notable progress in mental health service awareness and the delivery of AUD treatment; however, the needs of AUD caregivers are often overlooked, which warrants further exploration to improve the quality of life of caregivers, which in turn is related to patients’ recovery.

Ayurveda illustrates therapeutic success as a collective and relational process rather than a physician- or medicine-centric intervention. Ayurvedic texts describe *Chikitsa Chatushpada*—the *four indispensable pillars of treatment*—as essential for achieving optimal clinical outcomes. These pillars are *Vaidya* (the treating physician), *Paricharaka/Upasthata* (the attendant or caregiver), *Rogi* (the patient), and *Aushadha* (the medicine or therapeutic intervention) (6, 7). The harmonious presence and functional adequacy of these four are considered critical for successful healing.

Among these, the *Paricharaka* (caregiver) occupies a pivotal role in facilitating effective treatment. Ayurveda explicitly describes four essential qualities that a caregiver should possess: *Buddhiman* (intelligent), which reflects the ability to comprehend and accurately implement the physician’s instructions; *Daksha* (skilled, alert, and active), denoting efficiency, attentiveness, and practical competence in caregiving tasks; *Anurakta* (affectionate and emotionally balanced), signifying empathy, compassionate involvement, and emotional connectedness with the patient; and *Shuchi* (pure and clean), encompassing both physical hygiene and mental purity. Together, these attributes help create a supportive and healing environment (7).

These Ayurvedic qualities can also be viewed through the lens of psychological capital (PsyCap). *Buddhiman* contributes to realistic optimism and resilient decision-making; *Daksha* strengthens self-efficacy through competence and capability; *Anurakta* enhances emotional regulation, social connectedness, and caregiving motivation, while *Shuchi* promotes mental clarity and emotional balance, thereby reducing cognitive burden. Collectively, these qualities may foster PsyCap and contribute to improved psychological well-being (PWB) among caregivers facing sustained emotional and practical demands.

The concept of PsyCap is derived from Positive Psychology, which stated to enhance an individual’s capacity to manage care-giving related challenges and improve their overall functioning. The existing literature on PsyCap indicates that caregivers’ psychological resources significantly influence patients’ recovery and, conversely, patients’ health status impacts caregivers’ wellbeing (8). This reciprocal relationship signifies the importance of these resources in promoting caregivers’ PWB.

PsyCap, which defines it as an individual’s psychological resources, encompassing hope, self-efficacy, resilience, and optimism (9). Individuals with these psychological resources are more likely to engage positively with life’s challenges (10). Much of the literature on PsyCap has been associated with organizational behavior concepts such as job satisfaction, life satisfaction, commitment, stress, and psychological and physiological strain. In the context of mental health, most research on PsyCap has focused on the mental health of the general population (11), with only a few studies targeting caregivers of specific disorders, such as substance use (12) and Autism Spectrum Disorder (ASD) (13). This research indicates that enhancing PsyCap can lead to a decrease in work-related stress among service providers in drug abuse treatment clinics (14). Similarly, among parents of children with ASD, PsyCap mitigates stress among caregivers of ASD (13). Further, there is a need to understand how these four interrelated personal assets predict PWB among caregivers of individuals with AUD.

Psychological well-being is defined as engagement with the existential challenges of life. In 1995, Ryff proposed a six-dimensional model of PWB, which includes dimensions of purpose in life, autonomy, personal growth, environmental mastery, positive relationships, and self-acceptance (15). Studies have connected PWB to better mental health, resilience, decreased stress and illness and longevity (16, 17). Research published in the area of caregiving indicates that caregiving for a spouse is often correlated with increased levels of depression and a decline in overall wellbeing. Specifically, caregiving wives report diminished personal mastery, deteriorating positive relationships, and a reduced sense of purpose, self-acceptance, and autonomy (1). Similarly, research on African immigrant caregivers who believed in home care as a priority reported burden levels, indicating that cultural beliefs influenced PWB and stress levels of the caregivers (18). Longitudinal studies on caregivers’ PWB demonstrate that stable internal resources—such as a sense of control, personal mastery, and social support—facilitate personal growth and environmental mastery more effectively than individuals with fewer stable resources (19). Further assessing PWB among caregivers of individuals with AUD is crucial for understanding how they navigate the existential challenges related to caregiving.

A theoretical framework that can connect PsyCap and PWB among caregivers of individuals with AUD is the “conservation of resources theory.” Theory suggests that individuals aim to protect their psychological resources. Caregivers with higher PsyCap are better able to manage their stressors and emotional burdens associated with AUD (20). The integration of the Ayurvedic framework of *Chikitsa Chatushpada* with the contemporary model of conservation of resources underscores the notion that caregivers’ psychological resources are not merely ancillary but fundamental to therapeutic success and their wellbeing.

In India’s National Mental Health Programme, there is a significant focus on enhancing community mental health services and promoting active community involvement. Primary health care professionals act as the main platform for providing comprehensive and sustainable care for individuals with AUD and their families (21). This research examines the impact of PsyCap on caregivers’ PWB using a mixed-method approach. The goal of the study is to obtain deeper insights into how caregiver strengths and psychosocial stressors influence their wellbeing. This understanding aims to equip primary health care professionals with the knowledge necessary to enhance caregiver resilience and coping abilities, ultimately improving treatment adherence and recovery outcomes for patients.

## 2 Methodology

### 2.1 Objectives of the study

1. To assess the predictors of the PWB of family caregivers of individuals with AUD.
a. To identify sociodemographic variables as predictors of PWB.
b. To identify PsyCap as a predictor of PWB.
2. To explore the influence of psychosocial factors on PWB of caregivers of AUD.

### 2.2 Research design and sample size

**FIGURE 1:**
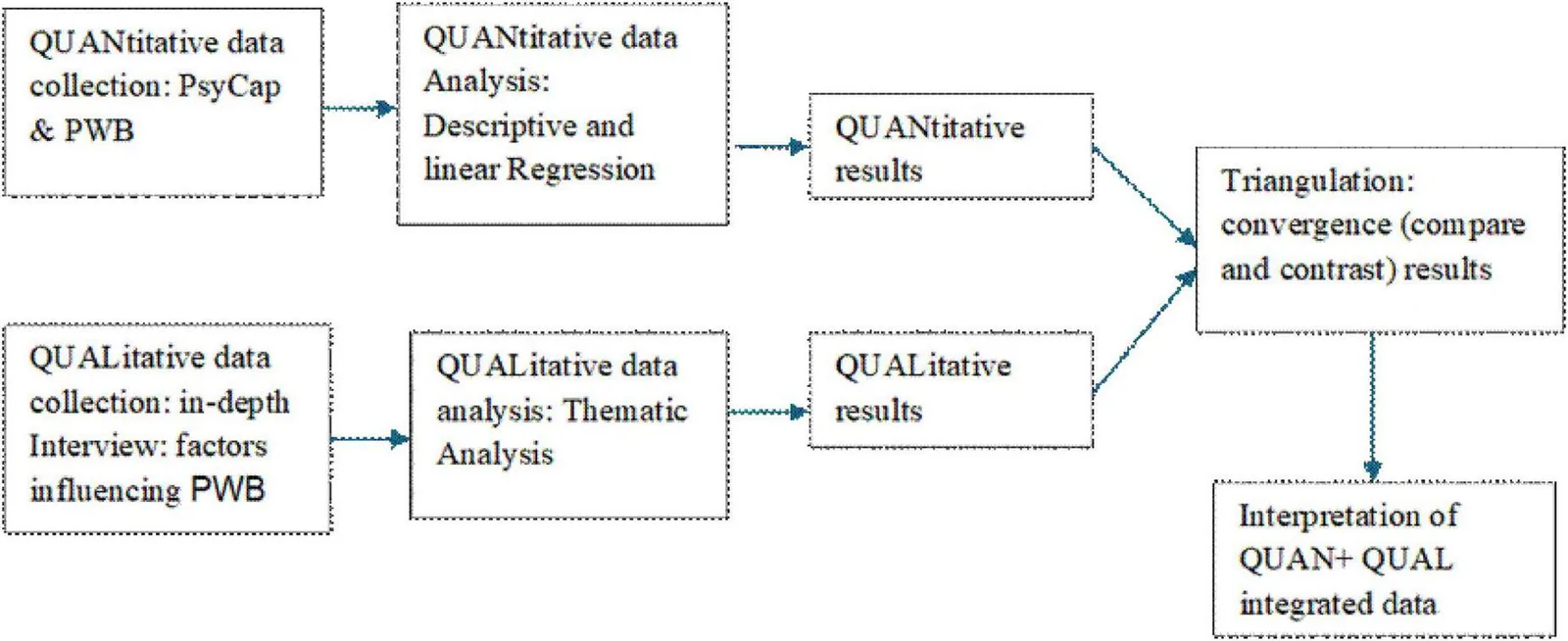
Concurrent convergence triangulation model.

A cross-sectional mixed study design with a concurrent convergence triangulation model was used. The data were collected from the Department of Psychiatry, Government Wenlock District Hospital, Mangalore, India. Qualitative and quantitative data were collected simultaneously, analyzed separately, and then compared and contrasted to give a joint interpretation. The research design used in the current study is presented in Figure 1.

### 2.3 Quantitative phase I

#### 2.3.1 Hypothesis of the study

***H_01_:*** Sociodemographic variables are not significant predictors of PWB.

***H_02_:*** PsyCap is not a significant predictor of PWB.

#### 2.3.2 Sampling method

A purposive sampling method was utilized to recruit caregivers of family members who were clinically diagnosed with alcohol use disorder and who had cohabitated with the patient for a minimum duration of 1 year. The caregivers, such as spouses, parents, siblings, adult children or other primary caregivers aged between 18 and 65 years, were included in the study. It excluded any participant whose patient had comorbid physical or psychiatric illnesses, if they were dependent on psychoactive substances other than alcohol, or if the participant had any physiological or psychiatric ailments.

#### 2.3.3 Sample size

Power analysis determined the sample size, with the estimated power as 0.84. The effect size was estimated using the objective. Based on the analysis, a sample size of 125 was determined.

#### 2.3.4 Tools used

Sociodemographic data sheet: This tool assessed various sociodemographic factors of caregivers, including age, sex, education, occupation, socioeconomic status, duration of coexistence with the patient etc.

Compound PsyCap scale (22): This scale comprises 12 self-evaluating statements covering four dimensions, such as hope, self-efficacy, resilience, and optimism. The participants were asked to rate his/her response on a six-point Likert scale; the higher the score, the greater the PsyCap. The scale has a Cronbach’s alpha coefficient of .82.

Ryff PWB scale (15): This 18-item self-administered scale comprises six factors, such as self-acceptance, environmental mastery, positive relationships, personal growth, purpose in life, and autonomy. The responses of the participants were asked to be rated on a seven-point Likert scale. The higher scores on the scale indicate greater wellbeing. The psychometric qualities of the scale were reported to be high.

#### 2.3.5 Translation of study tools

The materials utilized in this research study were translated into *Kannada*, the regional language, utilizing the back-translation method conducted by experts fluent in both English and *Kannada* to ensure the validity of the scale.

#### 2.3.6 Procedure

The data collection for the research was initiated after obtaining approval from the ethical committee. The family caregivers who accompanied patients with alcohol use disorders to hospitals were approached to participate in the study. The study purpose was explained to those who met the inclusion criteria. Written informed consent was obtained from participants who gave their consent to engage in the research. Based on their language preferences, participants were provided with either the *Kannada* or English version of the scales. They were instructed to complete the sociodemographic data sheet first, followed by the Compound PsyCap Scale-Revised and the PWB Scale last. The average duration for completing the questionnaire was between 10 and 15 min. For participants who experienced difficulty comprehending the items in the scale, the researcher read the questions aloud without altering the wording, resulting in an estimated total completion time of 20 min.

#### 2.3.7 Data analysis

In this study, PWB served as the dependent variable, whereas sociodemographic variables and PsyCap functioned as the independent variables. Jamovi version 2.4.1 software was used for statistical analysis. The study maintained standard confidence levels of 0.05, 0.01, and 0.001. The normality testing was carried out to analyze the normality of the data. Cronbach’s alpha was used to determine the internal reliability of the PsyCap and PWB scales. Further regression analysis was carried out to determine the predictors of PWB.

### 2.4 Qualitative phase II

#### 2.4.1 Research concern

To explore the influence of psychosocial factors on the PWB of caregivers of AUD.

#### 2.4.2 Sample size

Participants were purposively recruited, and semi-structured interviews were conducted with 10 caregivers of individuals with AUD.

#### 2.4.3 Tool used for the data collection

A semi-structured interview schedule was developed based on the existing literature by the researcher. It included items such as challenges of the caregivers, consequences on their lives, coping patterns, their belief systems, self-efficacy, etc., assessing the psychosocial factors that influence PWB of the caregivers.

#### 2.4.4 Procedure

The data collection for the qualitative research was conducted in the inpatient and outpatient departments of Government Wenlock Hospital, Mangalore. Participants who completed the questionnaires from the quantitative survey were asked to participate in the interview immediately after they filled out the questionnaires. Those who gave consent for the qualitative data collection were given a separate informed consent form and participant information sheet before initiating the interview. A semi-structured in-depth interview schedule of 30–40 min was conducted with an audio recording to assess the above objectives. Permission for the audio recording was taken separately, and if not given, the interviewer took down notes.

#### 2.4.5 Data analysis

Qualitative thematic analysis was utilized to evaluate the qualitative data. Interviews were transcribed and translated from Kannada to English. The transcribed data was analyzed using thematic analysis, following Sterling’s model (23). Initial codes were generated by using open coding, which were then categorized into basic themes. The basic themes were re-checked and organized further into organizing themes. Finally, global themes were found to explain the overarching trend in the data. Qualitative analysis experts were also consulted to cross-check the codes and themes that were produced from the study to ensure correctness and trustworthiness. The obtained results were then compared and contrasted with the quantitative data to give a joint interpretation.

## 3 Results

### 3.1 Quantitative analysis

#### 3.1.1 Normality testing

The total score distribution of PWB (Shapiro-Wilk statistic = 0.98, *p* = 0.32) was normally distributed, and PsyCap (Shapiro-Wilk statistic = 0.89, *p* ≤ 0.001) was not normally distributed. Hence, as one of the scales violated the normality assumption, non-parametric tests were chosen for carrying out further analysis. Table 1 presents tests of normality for psychological capital and psychological well-being.

**TABLE 1:** Descriptive statistics and normality testing.

Normality testing								
Variable	*N*	Mean	SD	Median	Skewness	Kurtosis	Shapiro wilk	*P*-value
PWB	125	78.9	12.7	79	−0.156	−0.163	0.988	0.325
PsyCap	125	30.8	12.6	26	0.993	0.135	0.894	0.001

PsyCap, psychological capital; PWB, psychological well-being.

#### 3.1.2 Internal reliability

An internal reliability test was conducted independently on the PsyCap and PWB. The analysis showed good internal consistency, with Cronbach’s alpha = 0.763 for PsyCap and 0.644 for PWB. An item-specific reliability showed that, based on the alpha values, none of the items needed to be dropped from the dataset. Tables 2, 3 present item-wise Reliability Statistics for PsyCap and for PWB.

**TABLE 2:** Item-wise reliability statistics for compound PsyCap scale revised (CPC-12R).

Internal reliability	
PsyCap scale items	If item dropped Cronbach’s α
I am looking forward to the life ahead of me.	0.750
Overall, I expect more good things to happen to me than bad	0.738
The future holds a lot of good in store for me.	0.715
I tend to bounce back quickly after serious life difficulties	0.739
Failure does not discourage me.	0.779
I consider myself a person who can withstand a lot.	0.759
I can remain calm when facing difficulties because I can rely on my coping abilities.	0.732
I can solve most problems if I invest the necessary effort.	0.740
I am confident that I could deal efficiently with unexpected events.	0.759
Right now, I see myself as being pretty successful.	0.749
I can think of many ways to reach my current goals	0.742
If I should find myself in a jam, I could think of many ways to	0.751
Cronbach’s alpha value for PsyCap	0.763

**TABLE 3:** Item-wise reliability statistics for Ryff’s psychological well-being scale (PWB).

PWB scale items	If item dropped McDonald’s ω
“I like most parts of my personality.”*	0.667
“When I look at the story of my life, I am pleased with how things have turned out so far.”*	0.722
“Some people wander aimlessly through life, but I am not one of them.”*	0.688
“The demands of everyday life often get me down.	0.680
“In many ways I feel disappointed about my achievements in life.”	0.662
“Maintaining close relationships has been difficult and frustrating for me.”	0.699
“I live life one day at a time and don’t really think about the future.”	0.686
“In general, I feel I am in charge of the situation in which I live.”*	0.685
“I am good at managing the responsibilities of daily life.”*	0.679
“I sometimes feel as if I’ve done all there is to do in life.”	0.679
“For me, life has been a continuous process of learning, changing, and growth.”*	0.718
“I think it is important to have new experiences that challenge how I think about myself and the world.”*	0.686
“People would describe me as a giving person, willing to share my time with others.”*	0.673
“I gave up trying to make big improvements or changes in my life a long time ago”	0.692
“I tend to be influenced by people with strong opinions”	0.688
“I have not experienced many warm and trusting relationships with others.”	0.685
“I have confidence in my own opinions, even if they are different from the way most other people think.”*	0.693
“I judge myself by what I think is important, not by the values of what others think is important.”*	0.692
Cronbach’s alpha for PWB scale	0.644

*Items are reverse scales items.

#### 3.1.3 Socio-demographic characteristics of the participants

The data presented in Table 4 provide an overview of the sociodemographic characteristics of the caregivers. A large proportion of the participants were female, accounting for 75% of the sample. The ages of the caregivers ranged between 18 and 64 years, with a mean age of 40.1 years and a standard deviation of 11.7. Additionally, 9.6% of the caregivers were classified as having a lower socioeconomic status. Among the caregivers, the familial relationships with the patients varied, 34.4% were spouses, 31.2% were parents, 14.4% were siblings, 5.6% were adult children, and 14.4% represented other categories of primary caregivers, including nieces, nephews, paternal uncles, cousins, and daughters-in-law. In terms of education, 43.2% of the caregivers had attained middle school qualifications, and 81% were employed. Furthermore, 68.8% of caregivers reported having provided care for over 10 years, while 25.6% had been caregivers for a duration of 1–5 years, and the remaining 5.6% had offered care for 5–10 years.

**TABLE 4:** Presents socio-demographic characteristics of the caregivers.

Socio-demographic variables	Categories	Percentage
Age	Young adults	52%
	Middle adults	42%
	Older adults	6%
Gender	Male	24.8%
	Female	75.2%
Education	Middle school (upto 7^th^ std)	53%
	High school 10^th^ std	17%
	Higher secondary 12^th^	13%
	Degree	9%
	Master/post-graduation	8%
Occupation	Employed	81%
	Unemployed	19%
Income	Upper social status	1%
	Middle social status	9%
	Lower social status	90%
Relationship status	Spouse	34.4%
	Parent	31.2%
	Sibling	14.4%
	Adult child	5.6%
	Other primary caregivers	14.4%
Duration of stay	1–5 years	25.6%
	5–10 years	5.6%
	>10 years	68.8%
Duration of alcohol use	1–5 years	56.0%
	5–10 years	24.8%
	>10 years	19.0%

#### 3.1.4 Descriptive analysis for PsyCap and PWB among caregivers

Descriptive statistics for caregivers’ PsyCap, as shown in Table 5, indicate that caregivers scored high on optimism (8.55) and resilience (8.10). This indicates that they tend to maintain a positive outlook and are able to bounce back from stressors. However, hope (6.98) and self-efficacy (7.14) are slightly lower, reflecting that caregivers feel less confident and hopeful in managing the caregiving demands. The mean total PsyCap score (30.3) is moderate relative to the possible range (13–68). This suggests that caregivers possess psychological resources at a moderate level. Stress and caregiver burden may have limited their full potential. Looking at PWB scores, which are reasonably high and consistent indicating high existential wellbeing. The mean and median are almost identical, suggesting a balanced distribution (median = 79, median = 78.9).

**TABLE 5:** Descriptive analysis of PsyCap and PWB among caregivers.

Variable	Median	Mean	SD	Minimum	Maximum
Hope	6	6.98	4.46	3	21
Optimism	8	8.53	4.46	3	21
Self-efficacy	7	7.14	3.75	3	21
Resilience	8	8.10	3.87	3	21
Total PsyCap	26	30.3	12.6	13	68
Total PWB	79	78.9	12.7	51	110

#### 3.1.5 Hypothesis testing

##### 3.1.5.1 *H_01_:* socio-demographic variables are not significant predictors of PWB

**FIGURE 2:**
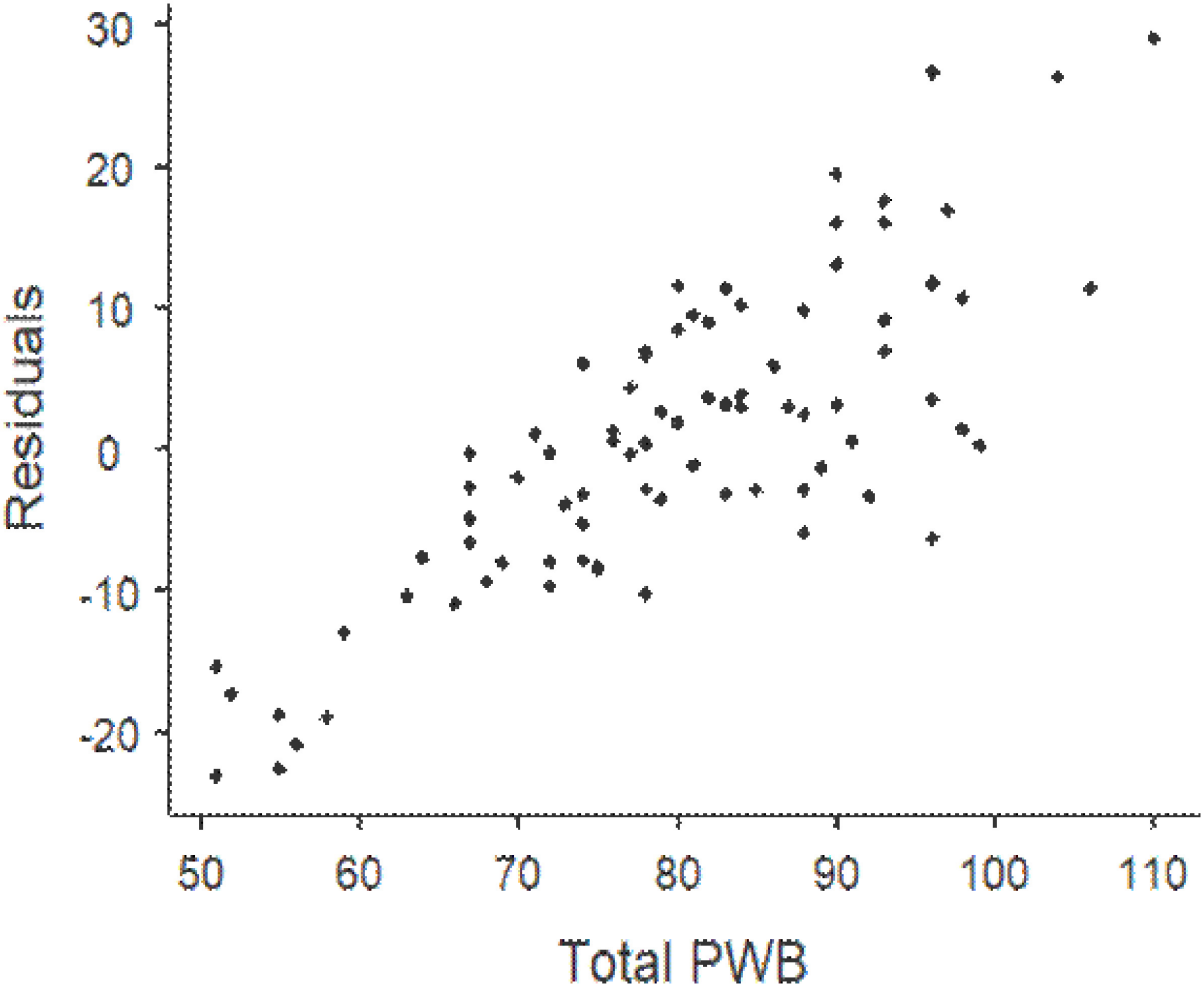
Homoscedasticity of the socio-demographic and psychological well-being (PWB) variables.

Prior to the regression analysis, assumption checks were conducted. The Durbin-Watson statistic indicated no evidence of autocorrelation; collinearity statistics revealed acceptable variance inflation factors (VIF ranging from 1.20 to 1.30) and tolerance above 0.70, suggesting no multicollinearity concerns. The Shapiro-Wilk test confirmed normality of residuals (W-0.1984, *p* = 0.137) visual representation of Figure 2 also indicates homoscedasticity, and Table 6 presents an assumption check for regression analysis.

**TABLE 6:** Assumption checks for multiple linear regression.

Durbin-Watson test for autocorrelation	Autocorrelation = −0.147 DW = 2.29	*P* = 0.126
**Collinearity statistics**	**VIF**	**Tolerance**
Age	1.20	0.83
Sex	1.30	0.76
Education Level	1.20	0.83
Occupation Status	1.20	0.83
Family Income	1.22	0.82
Relationship status with patient	1.21	0.82
Duration of stay with patient	1.22	0.81
Shapiro-Wilk Test for normality	Statistic = 0.984	*P* **=** 0.137

The regression results are presented in Table 7. The overall model is significant, with several predictors contributing to PWB. Sex was a significant predictor, with males reporting higher wellbeing compared to females (B = 6.15, SE = 2.92, t = 2.11, *p* = 0.03). Education was consistently significant, with higher education associated with greater PWB. Participants with a bachelor’s degree reported significantly higher PWB compared to illiterates (B = 22.31, SE 5.46, t = 4.08, *p* = 0.001). Similarly master’s degree holders also showed the association with PWB (B = 26.50, SE = 5.56, t = 4.76, *p* = 0.001). Occupation status was significant with unemployment individuals reporting lower PWB compared to employed individuals (B = −6.44, SE = 2.96, t = −2.18, *p* = 0.03). Relationship status with patient showed parents reported lower wellbeing compared to spouses (B = −6.20, SE = 2.92, t = −2.12, *p* = 0.03). The regression model explained 37% of the variance in PWB (R^2^ = 0.370), suggesting that sociodemographic factors of participants influence PWB; hence, the null hypothesis is rejected.

**TABLE 7:** Regression coefficient predicting psychological well-being (PWB).

Predictor	Estimate	SE	t	*P*
intercept	78.21	4.68	16.7	0.001
Age				
Young adults vs. middle adults	−3.15	2.57	−1.22	0.22
Old adults vs. middle adults	−2.45	4.52	−0.54	0.58
Sex				
Male vs. female	6.15	2.92	2.108	0.03
Education level				
Middle school vs. illiterate	2.72	3.19	0.85	0.39
SSLC vs. illiterate	9.54	3.92	2.43	0.01
PUC vs. illiterate	9.30	4.31	2.15	0.03
Bachelors vs. illiterate	22.31	5.46	4.08	.001
Master vs. illiterate	26.50	5.56	4.76	0.001
Occupation				
Unemployed vs. employed	−6.44	2.96	−2.18	0.031
Family income				
Lower Middle vs. lower	−1.906	2.46	−0.77	0.44
Middle vs. lower	3.572	4.50	0.79	0.42
Relations status with patient				
Parent vs. spouse	−6.200	2.92	−2.12	0.03
Sibling vs. spouse	−6.381	4.05	−1.57	0.11
Adult son vs. spouse	−2.006	4.78	−0.41	0.67
Other primary caretaker vs. spouse	−4.720	4.43	−1.06	0.28
Duration of stay with patients				
6–10 vs. 1–5 years	−0.132	5.61	−0.023	0.981
More than 10 vs. 1–5 years	−0.818	2.97	−0.27	0.783
Model	R = 0.608		R^2^ = 0.370	

##### 3.1.5.2 *H*_02_: PsyCap is not a significant predictor of PWB

**FIGURE 3:**
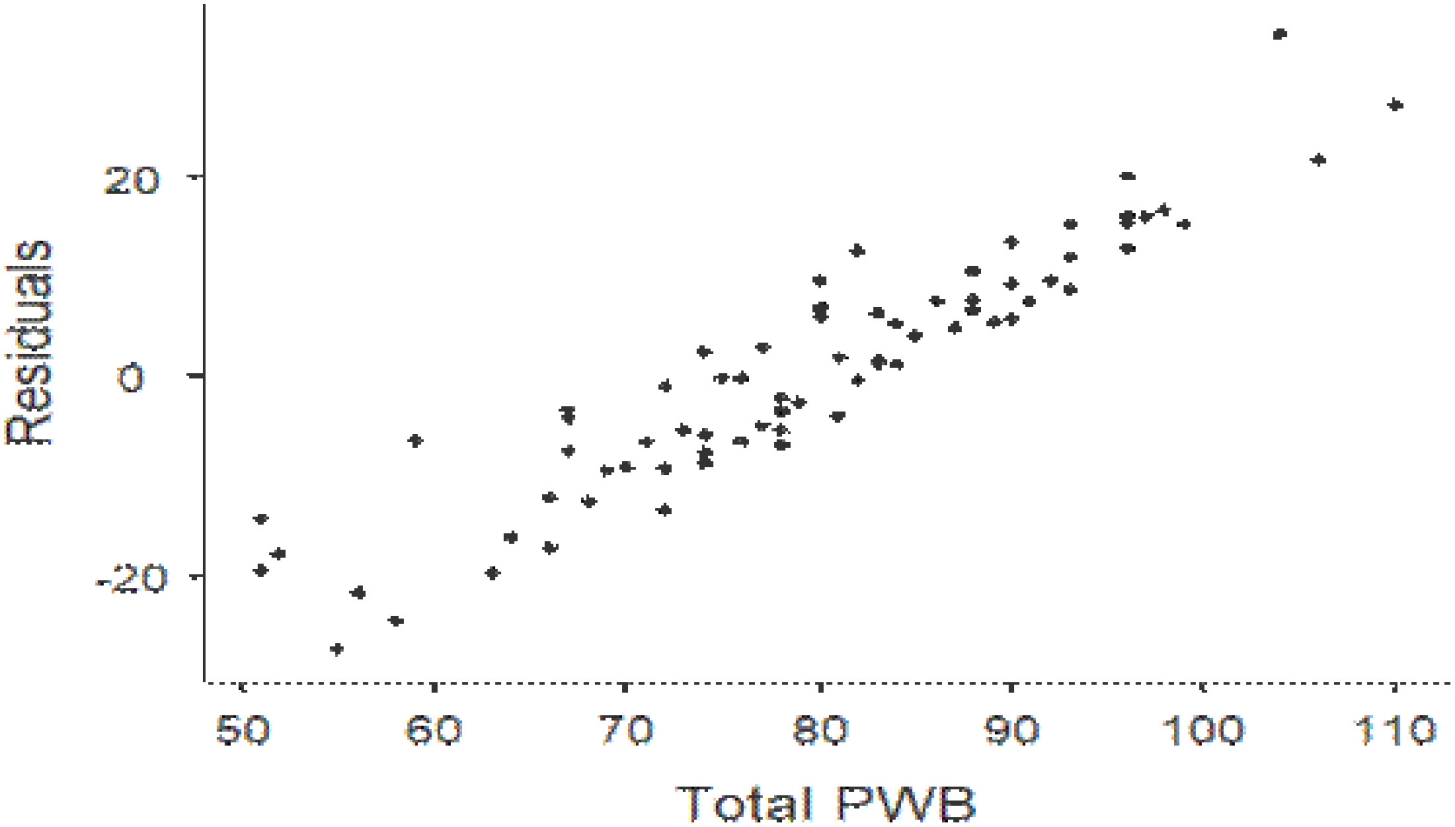
Homoscedasticity of the psychological capital (PsyCap) and psychological well-being (PWB) variable.

**FIGURE 4:**
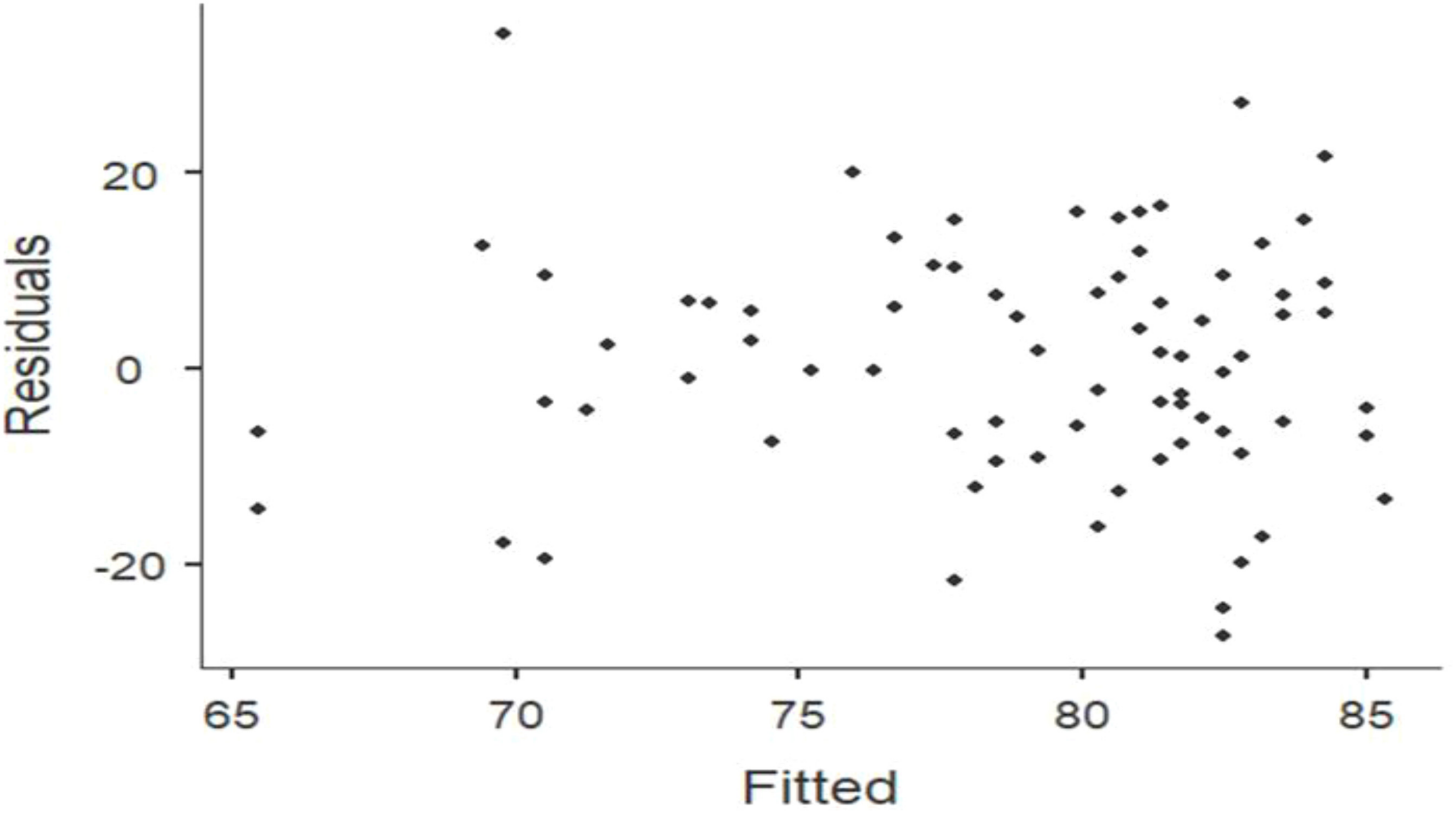
Residuals plot of the fitted model to determine collinearity of psychological capital (PsyCap) and psychological well-being (PWB).

A linear regression was constructed to test the hypothesis, stating that PsyCap is not a significant predictor of PWB. The study met the various assumptions of a Linear Regression model – observations are independent, as seen in the Durbin-Watson test for autocorrelation (DW = 2.03, *p* = 0.844); there exists a linear relationship between the PWB and the PsyCap (Table 8). Homoscedasticity is maintained as depicted in the residuals scatter plot (Figure 3); there exists no collinearity (VIF = 1, Tolerance = 1, Figure 4); and the residuals are approximately normally distributed. The Shapiro-Wilk test confirmed normality of residuals (W = 0.990, *p* = 0.469).

**TABLE 8:** Assumption check for linear regression for psychological capital (PsyCap) and psychological well-being (PWB).

Durbin-Watson Test for autocorrelation	Autocorrelation = −0.015 DW = 2.03	*P* = 0.844
Collinearity statistics	VIF	Tolerance
Total PsyCap	1	1
Shapiro-Wilk Test for normality	Statistic 0.99	*P* = 0.46

Tables 9, 10 present regression analysis for PsyCap and PWB among caregivers. The PsyCap explained a significant 13% of the variance in PWB (R^2^ = 0.129, F = 18.2, *p* ≤ 0.001), indicating a good fit. Therefore, as PsyCap is a significant predictor of PWB, the study rejects the null hypothesis H_02._ The regression coefficients are presented in Table 10; total PsyCap was a significant negative predictor of PWB. Lower PsyCap scores were associated with high PWB in this group (B = −0.361, SE = 0.085, t = −4.27, *p* < 0.001). Despite depleting psychological resources, caregivers could maintain high existential wellbeing, which is also evident in the descriptive statistics presented in table number 5.

**TABLE 9:** Standard linear regression results.

Variables	R	R^2^	*F*	*P*
Total PsyCap	0.359	0.129	18.2	<0.001

**TABLE 10:** Regression coefficients predicting psychological well-being (PWB).

Predictors	Estimate	Std error	t	*P*
Intercept	90.023	2.8101	32.04	<0.001
Total PWB	−0.36	0.0846	−4.27	<0.001

### 3.2 Qualitative findings

Table 11 presents a sociodemographic profile of the 10 study participants who were interviewed to understand psychosocial factors influencing PWB of the caregivers. The sample included a predominantly female cohort (*n* = 8), with ages ranging from 18 to 62 years. The majority of participants were spouses of the patients, (*n* = 7), while two were parents and one was an adult child. Education levels varied from illiterate to senior secondary school, though most had completed middle or higher secondary schooling. Economically, all participants belonged to the lower socioeconomic status (SES), with 70% being employed and 30% unemployed. Notably, every participant reported a long-term cohabitation period with the patient exceeding 10 years,

**TABLE 11:** Socio-demographics of the qualitative sample.

Participant no	Age	Sex	Education level	Occupation status	Family income	Relationship status with patient	Duration of stay with patient
P01	44	F	Higher secondary	Unemployed	Lower SES	Spouse	>10 years
P02	62	M	Middle school	Unemployed	Lower SES	Parent	>10 years
P03	30	F	Middle school	Employed	Lower SES	Spouse	>10 years
P04	30	F	Higher sec school	Employed	Lower SES	Spouse	>10 years
P05	35	F	Higher sec school	Employed	Lower SES	Spouse	>10 years
P06	28	F	Middle school	Employed	Lower SES	Spouse	>10 years
P07	48	F	Higher sec school	Employed	Lower SES	Spouse	>10 years
P08	32	F	Middle school	Employed	Lower SES	Spouse	>10 years
P09	60	M	Illiterate	Employed	Lower SES	Parent	>10 years
P10	18	M	Senior secondary school	Unemployed	Lower SES	Adult child	>10 years

**FIGURE 5:**
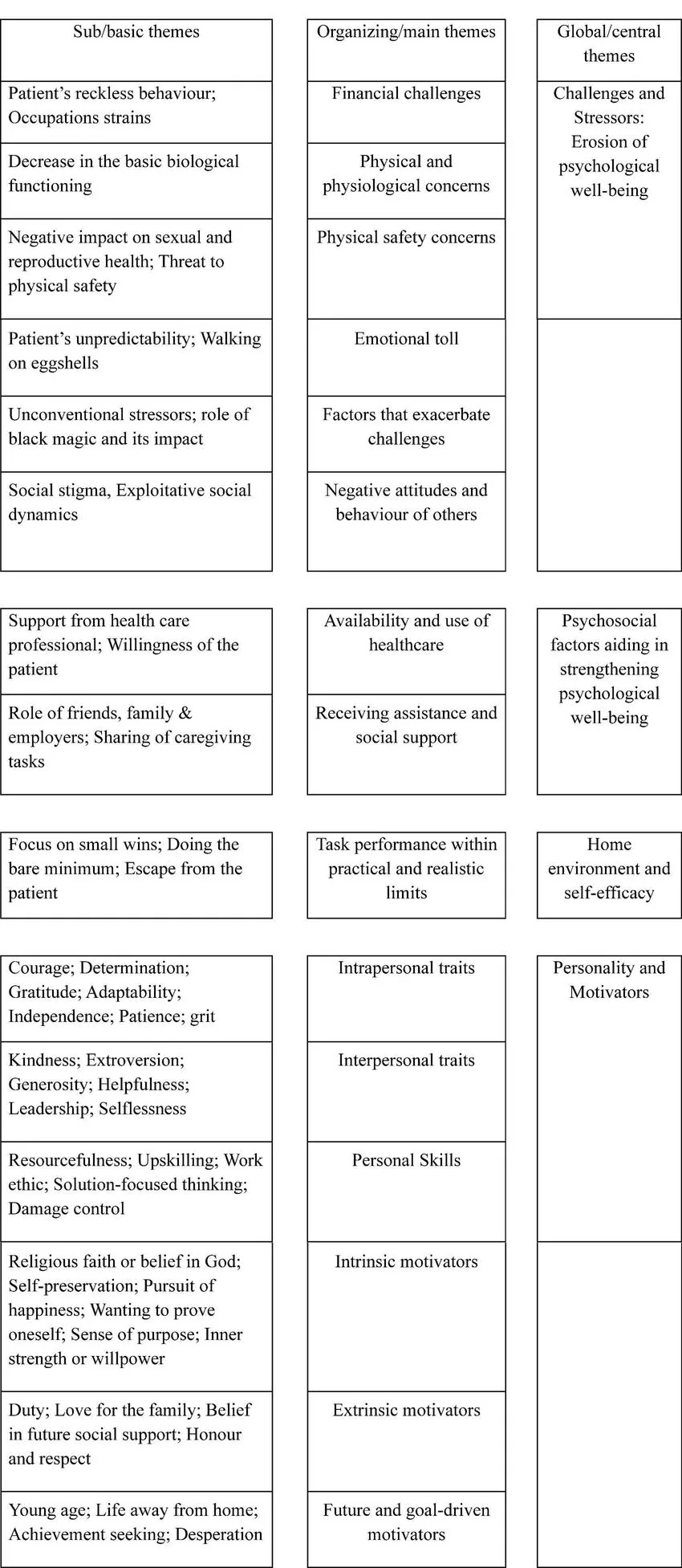
Summarizes the main findings of the qualitative phase.

In the current study, Stirling’s thematic network was used as a tool for conducting thematic analysis. The study’s findings gave way to five thematic networks, each of which will be discussed in further detail. Each thematic network consists of a central umbrella title called the Global theme or Central theme, which encompasses various Main or Organizing themes and Basic or sub-themes. Figure 5 summarizes the main findings of the qualitative phase. The following sections further illustrate and explore each thematic network.

#### 3.2.1 Global theme: challenges and stressors

The global theme addressed the multitude of challenges that caregivers faced, starting with the financial strain caregivers experienced. The economic burden was compounded by the patients’ problematic behavior such as reckless spending and stealing, which forced caregivers to look for added sources of income, borrow loans and delay retirement. Extracts such as “The biggest challenge is the financial difficulty. I’m working very hard as a cleaner in a hotel, but it’s barely enough to cover the basic expenses, let alone the crazy medical bills and the extra mess his drinking creates.” (P 6), highlight the same. This was further exacerbated due to job insecurity and the extended hours spent in caregiving.

The next sub-theme highlights the physical and physiological concerns that decrease the caregivers’ basic biological functioning. Increased fatigue, headaches, body aches, stress, reduced sleep, decrease in body weight and reduced self-care were common across caregivers. *“I also feel like I am always tired because I am either working, or dealing with my son, or coming to the hospital, or something or the other. No rest only. Because of this my weight also keeps going down” (P 8)* highlights the same.

Caregivers also faced unique physical safety challenges, including threats to their physical security and negative impacts on sexual and reproductive health which are compounded by the poor attitudes and behaviors of others. A total of 50% of the female participants had poor pre- and post-partum care due to added caregiving responsibilities which further reduced the acceptance of their own limits, and their freedom. *“When I delivered my first child it was very difficult. I had no one to help. I delivered my child, picked him up, went home, and within a week I started working. My husband continued drinking” (P 2).* Additionally, they had a reduced desire to engage in sexual intercourse with the patient, yet, they continued to experience unwanted sexual advances from the patient, which further compromised their safety. *“When he (patient) drinks and comes also he touches me uncomfortably. If I say no then he starts hitting me” (P 4).*

The next sub-theme highlights negative emotions caregivers faced due to the patient’s unpredictability. The emotional burden caregivers carried was immense, marked by feelings of anger, anxiety, sadness, and disappointment due to the patient’s negative interpersonal interactions. “I am always worried and irritated…If I see him there’s sadness because I love him but he struggles with this problem, and it hurts when you can’t make things better. It’s a lot of emotions. I feel angry, disappointed, and sometimes, moments of hope that things might change. It’s draining, to be honest.” (P 10). They walked on eggshells around the patient in order to avoid triggering an emotional outburst, which rooted a sense of constant vigilance and anxiety in the caregiver, thus reducing their positive interactions, and growth.

Caregivers also faced unconventional stressors that added layers to their challenges, such as cultural beliefs in black magic and systemic barriers that prevented them from leaving harmful situations. *“Near the village side the obstacles are very different… Mata mantra (black magic), religious ceremonies, many different things. They do all that. It won’t come into your psychology. We keep going to temples and sages asking them to improve our lives, spending all our money on them.” (P 1).*

The next sub-theme addressed the nature of the attitudes and behavior of others toward the caregiver, and its impact on them. Extracts such as *“There’s a lot of judgment and people talk behind our back when it comes to personal problems. Instead of support, they say bad things about us. Sometimes they even tell us to go die in the gutter. It’s hard to seek help or talk openly about the challenges we’re going through. People tend to blame me for his addiction” (P 5)* highlight the critical comments, scrutiny and judgment that caregivers faced. Moreover, 60% of the caregivers reported that neighbors, employers, and relatives would gossip, trivialize the struggles and blame the caregiver for the patient’s condition, further isolating the caregiver.

A unique finding of this study was the exploitative behavior of others, and the internalization of the societal attitudes by the caregivers. “Other people also think they can touch me because my husband is not good. Even when they ask me if I have any problems, if I need any money, they ask me with a very different purpose in mind… They all just want my body” (P 4). These themes reflected helplessness, hopelessness and reduced self-efficacy to handle effectively the challenges. Extracts such as “He often blames me for his problems. His words sometimes make me feel like a failure… He tells me that I am only the reason that he drinks so much. I have no self-confidence anymore to leave him also. What will I do, where will I go? I have no worth anymore” (P 6) highlight the same. The lack of social support and social stigma further exaggerated the financial, physical and emotional challenges as illustrated above which inversely affected on their PWB.

#### 3.2.2 Global theme: psychosocial factors aiding in facing challenges

Within the subject of positive psychology, the second global theme was crucial as it illustrated the psychological factors that strengthen the caregivers’ abilities, wherein healthcare access and some extent of social support emerged significant. Alternative medicine *“I even took him to some temples and to a priest for treatment. Like a Shaman you know? I’m always trying to find a way” (P 10)* and specialized medical attention *“Even now I have a lot of confidence that we can come out of this situation. The doctors are somehow giving me confidence” (P 1)* were emergent factors which instilled hope and optimism in the caregivers and reflected the importance of the patient’s willingness to seek such treatments – “*My husband, he doesn’t say no; doesn’t say he won’t do it. He does whatever I tell him to do. He only wanted to come to the hospital for treatment.” (P 1).* Thus, the caregiver’s ability to handle everyday responsibilities instilled hope.

Receiving few assistance and social support was a subtheme wherein the practical, emotional, and moral support received from social bonds reduced the sense of isolation caregivers experienced. It enabled effective navigation of caregiving roles and promoted resilience in them. *“I used to work in an ice cream shop. I called them up and told them that my husband was admitted, I had no food at home, and my son was just born. I am in a lot of trouble. She got me groceries for a whole month. Only after that, I was able to slowly get back on my feet.” (P 3)*. The sharing of caregiving responsibilities within the family further enhanced caregivers’ sense of freedom of choice, as illustrated in the extract *“My mother has been the biggest support system… I also try to help her everywhere so that she can also go to work and take a break from the house. So we are a team in managing my father and the house” (P 9)*.

#### 3.2.3 Global theme: home environment and self-efficacy

The global theme highlighted the factors in the home environment that relatively promoted the caregivers’ self-efficacy. Performing tasks within practical and realistic limitations was a prominent sub-theme wherein caregivers focused on small wins as a positive strategy. Celebrating incremental achievements, finding tranquility on days when there are no emotional outbursts at home, and experiencing happiness when spending time away from the patient, and with other family members enhanced their belief in their abilities. Extracts such as *“… just sitting and eating food with my mother, asking her about her day and telling her about my day is nice. It really makes me feel like it’s just the two of us in the house and that someday both of us will become successful away from my father (patient)…” (P 9)* highlight the same.

Caregivers also focused on doing the bare minimum instead of putting in their complete effort, especially for caregiving tasks. *“On most days I just focus on paying the bills, cleaning the mess at home, and making sure there’s food on the table. I do only what is 100% required” (P 8).* Hence, they set practical limitations for themselves under realistic expectations, which helped them navigate their role within manageable boundaries.

#### 3.2.4 Global theme: personality and motivators

The sub-theme of intrapersonal traits, such as courage, determination, gratitude, adaptability, independence, patience and grit “I’ve always been kind of stubborn, which helps me keep going even when things get tough. It’s like this survival instinct kicking in… There’s this resistance to letting the difficulties define my entire existence.” (P 6). Caregivers characterized by higher levels of these traits exhibited a sense of hope and optimism concerning their future, as these traits served as a reliable safety net and enabled caregivers to persist for longer by delaying the negative effects of caregiving.

The next sub-theme of interpersonal traits was tightly linked with the caregivers’ social dynamics. Traits such as kindness, helpfulness, leadership and selflessness endowed caregivers with the ability to face challenges. These traits aided the caregivers in establishing collaborative networks with other family members, leading the household during difficulties, and putting the wellbeing of others ahead of theirs. The outcome was strengthened relationships, which in turn increased the guidance, assistance and support that caregivers received. *“I am very selfless also, I never care about myself, only about other people. Because of this only I am able to sustain a relationship with my husband’s uncle who is helping us so much.” (P 8).*

Caregivers also showcased the sub-theme of personal skills, which supported their ability to effectively deal with their challenges. Qualities such as resourcefulness, propensity for upskilling, strong work ethic, solution-focused thinking, and the capacity for prompt damage control in challenging circumstances emerged as favorable as they helped them with economic, social and pragmatic challenges. “I am naturally like a leader, because I am the head of the family and everyone is dependent on me. So because of this at work also people respect and value me a lot and at home also. So because of this, even today in society, our family name has not gone down too much.” (P 8).

The sub-theme of intrinsic motivators seemed to maintain the caregiver’s resilience and optimism. Factors such as utilizing religious faith or belief in God, self-preservation, pursuit of happiness, wanting to prove oneself, a sense of purpose and an indescribable inner strength or willpower allowed caregivers to exhibit an overcommitment to their role and foster a mindset in the face of adversity. *“I get the motivation in my mind itself. I tell myself “God will help me, he will make sure I move on and come out of this”. This gives me courage.” (P 2, 5).*

Extrinsic motivators such as perceiving caregiving as a familial duty, love for the family, belief in future social support, and pursuing honor and respect in society emerged as significant. A total of 70% of the caregivers felt compelled to persevere due to having dependent children, which empowered them to endure extended periods of caregiving, as illustrated in the extract “Only my children motivate me to keep going and see what will happen in the future. But I am sure my children will stand on their own feet and make a good future for me as well” (P 4). Therefore, the unwavering support, encouragement and familial bonds from external sources instilled overcommitment and a depletion in their psychological resources despite experiencing high existential wellbeing as denoted in PWB.

Lastly, future and goal-driven motivators maintained their hope and self-efficacy. A total of 20% of the caregivers believed their youth afforded them sufficient time to address the patient’s concerns and continue leading their lives, *“I’ve got this stubborn hope that drives me. I want to break this cycle. I dream about a future where I’m not hiding bottles or worrying about what mood he’s (patient) in. That vision of a better life keeps me pushing through this.” (P 10).* As a last resort, 20% of caregivers exhibited a sense of desperation to find solutions, utilizing optimism as a motivator in itself, driven by the belief that they had no choice but to persist and move forward. Consequently, future-oriented and goal-driven motivators instilled some sense of purpose, provided self-confidence by instilling hope, optimism and self-efficacy in the caregivers.

## 4 Discussion

### 4.1 Socio-demographics as a predictor of PWB

The present study aimed to examine the predictors of PWB among primary family caregivers of individuals diagnosed with AUD. The findings indicated that sociodemographic variables moderately served as significant predictors of PWB. Specifically, being male, having a high level of education, being employed, and being a spouse rather than a parent as a caregiver were identified as significant predictors of PWB among caregivers. Previous studies have reported that female caregivers often experience lower PWB than their male counterparts (24, 25). Additionally, *Bharti* found that a higher level of education and paid employment were significant predictors of wellbeing among caregivers of patients with psychiatric illnesses (26).

### 4.2 PsyCap as a predictor of PWB

Previous studies in the general population found that PsyCap predicts 12% of PWB. Similarly, the current study identified a 13% predictive relationship between PsyCap and PWB among caregivers of individuals with AUD. However, the negative relationship observed indicates a paradoxical effect of PsyCap on PWB among these caregivers. A lower level of psychological resources among caregivers is associated with higher levels of caregiver distress and burden (27). It is well-documented that caregivers of individuals with AUD often experience moderate-to-severe caregiver burden (28). Several factors influence these burdens, including frequent hospital admissions, the amount of alcohol consumed by the individual in recovery, monthly expenditures on alcohol, and the presence of stigma, prejudice, and discrimination (29). As a result, caregivers experience resource depletion, where even strong psychological resources like optimism and resilience may become overextended, leading to burnout despite the fact that caregivers could experience high existential wellbeing, which is reflected through PWB parameters of personal growth, environmental mastery, purpose in life and autonomy. Various studies have highlighted that caregiver burnout has a significantly adverse impact on caregivers’ resilience, self-efficacy, and hope (30, 31). This phenomenon is also observable in the current study, where participants demonstrated moderate levels of psychological capital (PsyCap) while having high PWB. Furthermore, this relationship is further elucidated through the themes identified in the qualitative findings.

### 4.3 Integration of quantitative and qualitative findings

The current quantitative results indicate that 36% of the variance in PWB among caregivers can be explained by sociodemographic factors. However, there are many other factors that contribute to the remaining 64% of unexplained variance in wellbeing. Qualitative themes such as psychosocial support, access to healthcare, and positive personality traits were some of the factors identified as influencing caregiver wellbeing. Conversely, it was found that PsyCap is inversely related to the PWB of caregivers. The qualitative analysis revealed several additional factors, including external challenges, as well as both intrinsic and extrinsic motivations of the caregivers. These motivations encompassed religious faith, self-preservation, willpower, upskilling, solution-oriented thinking, and affection for their families. Although these factors contributed to a sense of overcommitment, which in turn depleted their psychological resources, in the long run they ultimately fostered higher levels of environmental mastery, personal growth, a sense of purpose in life, and autonomy, resulting in high overall psychological wellbeing. This is because of the quality of the caregiver mentioned in Ayurveda as *Anurakta* i.e., being affectionate and emotionally engaged, which makes them face the adversities and experience greater existential wellbeing.

Previous qualitative studies have found similar challenges faced by caregivers, such as financial (32), physiological (2), safety concerns (8), emotional burden (33), and negative societal attitudes (32, 34), however, the current study though mixed method design, highlights, along with external challenges, how intrinsic motivators led to increased existential wellbeing among caregivers of AUD. From the perspective of Conservation of Resources (COR) Theory, caregivers experienced significant depletion of psychological resources due to chronic stressors such as financial burden, emotional distress, stigma, and caregiving demands. Nevertheless, qualitative findings revealed that alternative resources, such as religious faith, willpower, family commitment, self-preservation, and solution-oriented coping, enabled them to continue investing in valued life. Although this commitment reduced PsyCap, it simultaneously fostered greater personal growth, environmental mastery, autonomy, and a strong sense of purpose in their life. Consequently, caregivers maintained high existential well-being despite lower levels of psychological capital. The study findings reflect the complexity of resource conservation among caregivers facing persistent adversity related to caregiving of individuals with AUD, highlighting more studies to understand the dynamic relationship between PsyCap and PWB among caregivers of individuals with AUD.

### 4.4 Implications

The study’s findings have both immediate and long-lasting implications. It is essential to assess the mental health of caregivers to prevent a decline in their wellbeing. Strengthening their internal resources, such as PsyCap, through targeted interventions is crucial for promoting their wellbeing. Additionally, social support groups and efforts to reduce stigma are important external resources that can help empower caregivers dealing with AUD. Professional support can be strengthened by integrating a holistic treatment team in primary health centers, which includes social workers and clinical psychologists. Further, promoting community awareness programmes and educating the public about the challenges, stigma, and mental health issues associated with AUD can improve societal perceptions and attitudes toward both caregivers and patients. This, in turn, can lead to better support systems in the long run.

## 5 Conclusion

This study focused on exploring the integration of the Ayurvedic concept of *Chikitsa Chatushpada* with established positive psychology concepts of PsyCap and PWB. It examined the predictors of PWB while exploring the psychosocial factors influencing wellbeing among caregivers of patients with AUDs. The research findings indicate that socio-demographic variables predict 35.9% of PWB, while psycap contributed 13% to the inverse relationship with caregivers’ PWB. Additionally, the qualitative data highlight themes related to associated challenges and stressors, as well as psychological factors that aid in facing these challenges. These study findings will enhance the in-depth knowledge of health care professionals in providing quality service to individuals with AUD and their caregivers.

## 6 Future scope and limitations

Although the study’s sample size was adequate for statistical analysis, it may limit the generalizability of the findings, particularly regarding the qualitative data. The focus on AUD restricts the transferability of the findings to other caregiving contexts. Gathering additional information about the quality of the caregiver-patient relationship, the types of social support received, access to healthcare, and the duration and number of treatments utilized are additional valuable socio-demographic details that can be considered in future research. Examining mediators such as burnout, role overload, and social support to clarify when PsyCap helps or harms wellbeing among caregivers of AUD. Additionally, future studies could benefit from a longitudinal design or intervention-based approach to assess how PsyCap and wellbeing evolve within the caregiving journey.

## Data Availability

The datasets presented in this study can be found in online repositories. This data can be found here: https://osf.io/wcbz4/overview?view_only=55e6b57f76154ded976f1d4aa3b2bf69.
